# Supplementary material for: Content-rich biological network constructed by mining PubMed abstracts
Source: BMC Bioinformatics. 2004 Oct 8;5:147. doi: 10.1186/1471-2105-5-147 (PMC528731; doi:10.1186/1471-2105-5-147)
Supplement: Additional File 5 — The original Chilibot query results of the term "long-term potentiation (LTP)" and 22 other terms, limiting the latest references analyzed to the years 1990, 1995, 2000, and 2004. [file 1471-2105-5-147-S5.bz2 › chilibotAdditionalFile5/ltp2004/html/TRKB_PKC.html]

 


 **TRKB** and **PKC** 
  
Found 7 abstracts in PubMed,  **7 abstracts were retrieved and analyzed**.  


---

 Search Google  |
 PDF files only 
|  EDU domain only 

---

**Interactive relationship** (e.g. stimulation, inhibition, etc)

- Our results suggest that  **PKC**  activation leads to a decrease in retinal cells proliferation through the release of acetylcholine and brain derived neurotrophic factor in the culture, and activation of M1 and  **TrkB**  receptors, respectively.  Ref: 12441170 Neurochem Int, 2003
- From these results we conclude that BDNF increases spontaneous network activity by suppressing GABAergic inhibition, the site of action of BDNF is predominantly postsynaptic, BDNF induced suppression of GABAergic synaptic transmission is caused by acute downregulation of GABA A receptors, and BDNF effects are mediated by its  **TrkB**  receptor and require  **PKC**  activation in the postsynaptic cell.  Ref: 12163512 J Neurophysiol, 2002

**Parallel relationship** (e.g. studied together, co-existance, homology, etc.)

- We labeled normal and nob retinas with antibodies against mGluR6,  **PKC** , G0alpha, bassoon, PSD 95, the alpha1F subunit of voltage gated calcium channels,  **trkB** , and dystrophin.  Ref: 14570248 Vis NeurosciVis Neurosci, 2002
